# How Ladies Should Dress

**Published:** 1864-04

**Authors:** 


**How Ladies should Dress.**—As you look from your windows in Paris, observe the first fifty women who pass; forty have noses depressed in the middle, a small quantity of dark hair, and a swarthy complexion. But, then, what a toilet! Not only suitable for the season, but the age and complexion of the wearer. How neat the feet and hands! How well the clothes are put on, and more than all, how well they suit each other!

Before English women can dress perfectly, they must have the taste of the French, especially in color. One reason why we see colors ill-arranged in England is that the different articles are purchased each for its own imagined virtues, and without any thought of what is to be worn with it. Women, while shopping, buy what pleases the eye on the counter, forgetting what they have at home. That parasol is pretty, but it will kill, by its color, one dress in the buyer's wardrobe, and be unsuitable for the others. To be magnificently dressed costs money; but to be dressed with taste is not expensive. It requires good taste, knowledge, and refinement. Never buy an article unless it is suitable to your age, habit, style, and the rest of your wardrobe. Nothing is more vulgar than to wear costly laces with a common delaine, or cheap lace with expensive brocades.

What colors, it may be asked, go best together? Green with violet; cold with dark crimson, or lilac; pale blue with scarlet; pink with black or white; and gray with scarlet or pink. A cold color generally requires a warm tint to give life to it. Gray and pale blue, for instance, combine well, both being cold colors. White and black are safe wear, but the latter is not favorable to dark or pale complexions. Pink is to some skins the most becoming; not, however, if there is much color in the cheeks and lips, and if there be even a suspicion of red in either hair or complexion. Peach color is, perhaps, one of the most elegant colors worn. Maize is very becoming, particularly to persons with dark hair and eyes. But whatever the colors or materials of the entire dress, the details are all in all;. the lace around the bosom and sleeves, the flowers—in fact, all that furnishes the dress. The ornaments in the head must harmonize with the dress. If trimmed with black lace, some of the same should be worn in the head, and the flowers, which are worn in the hair, should decorate the dress.—*All the Year Round.*

